# Traditional food taboos and practices during pregnancy, postpartum recovery, and infant care of Zulu women in northern KwaZulu-Natal

**DOI:** 10.1186/s13002-021-00451-2

**Published:** 2021-03-20

**Authors:** Mmbulaheni Ramulondi, Helene de Wet, Nontuthuko Rosemary Ntuli

**Affiliations:** Department of Botany, University of Zululand, Private bag X1001, KwaDlangezwa, Richards Bay, 3886 South Africa

**Keywords:** Dietary restrictions, Cultural beliefs, Pregnancy, Infant-care, Postpartum, Northern Maputaland, Food practices, Zulu women

## Abstract

**Background:**

Traditional practices and beliefs influence and support the behavior of women during pregnancy and childbirth in different parts of the world. Not much research has been conducted to examine whether and how cultural traditions continue to shape maternity experiences of Zulu women. The aim of this study is to establish the extent at which women in certain rural communities adhere to traditional food taboos and practices during pregnancy, postpartum recovery, and infants feeding, in comparison to what is recommended by health care workers.

**Methods:**

A survey was conducted in the rural northern KwaZulu-Natal between 2017 and 2020. A total of 140 women between the ages of 18 and 90 years were interviewed and they were chosen purposively based on their experiences in pregnancy, postpartum recovery, infant care, and their willingness to share the knowledge. Data were analyzed using descriptive statistics.

**Results:**

Most (64%) of the participants said that they adhered to these cultural food taboos and practices. The most common foods avoided were certain fruits [mango, naartjie, orange, papaya, and peach], butternut, eggs, sweets (sugar, commercial juice, sweet food, and honey), chili, ice, and alcohol. The most recommended foods during pregnancy were leafy vegetables, fruits (except the avoided ones), liver, and fish. For postpartum recovery, women mostly consumed soft porridge, all fruits and vegetables, beetroot, and tea. Food not allowed for children younger than 2 years included meat, sugar and sweets, and chewable foods.

**Conclusion:**

Differences on food taboos and practices between participants who received formal education and those who did not received it were insignificant. The beliefs about the detrimental effects of some foods were not backed up by scientific research. Restriction of some orange/yellow colored fruits during pregnancy that are rich in vitamin A and/or C may affect daily requirements of these micronutrients, and the foods recommended during pregnancy and postpartum period would not provide all the essential nutrients required for successful pregnancy. However, some of the food taboos would protect women from unhealthy eating. Our findings provide a basis for developing culturally appropriate nutritional mediation programs for Zulu women with a view to provide effective nutritional counseling.

## Background

Pregnancy requires a healthy diet that includes an adequate intake of energy in the form of proteins, vitamins, and minerals to meet maternal and fetal needs [1]. A healthy diet includes a variety of foods, such as green and orange vegetables, meat, fish, legumes, nuts, whole grains, and fruits [2]. A caloric intake of a pregnant woman should increase by approximately 300 kcal/day [3]. However, some pregnant women often lack access to a healthy diet that provides for their increased nutritional requirements because of some food taboos which are often practiced in low- and middle-income countries [4–6]. Food taboos are food and beverages that people abstain from consuming for religious, cultural, or hygienic reasons [7]. Several studies from Asia [4, 5, 8–10] and Africa [6, 11–14] have indicated that women from various parts of the world, during pregnancy and postpartum period, are forced to abstain from nutritious foods as part of their traditional beliefs. Evidence revealed that these food taboos accounts mostly to maternal and fetal malnutrition during pregnancy [15]. Inadequate intake of micronutrients may lead to different types of malnutrition, such as deficiencies of iron, vitamins and zinc, where insufficient vitamin A and iron-deficiency anemia contribute to at least 18% of maternal deaths in developing countries [16]. In many cultures, the belief is that the avoidance of certain food intake (food taboos) protects the health of the mothers and their unborn babies [7, 8, 13]. However, this may increase the risk of deficiency of proteins, fats, vitamin A, calcium, and iron in pregnant women [17].

Food taboos have a big influence on pregnant women as they have been followed by many generations and they also form part of their culture [6]. Consequently, people practicing these pregnancy-related taboos believe that breaking them may harm the unborn baby or threaten the health of the mother [18]. Additional reasons for women to avoid certain foods include their fear for possible miscarriages and difficulties during delivery [19]. Some women observe these food taboos because of their own previous experience or that of other women. Other women observe these taboos because it is a symbol of respect for elders [20]. This avoidance of food usually does not conform to the medical view about the appropriate types and quantity of foods needed by pregnant women to safeguard ideal maternal-fetal nutrition [21]. Traditional beliefs may influence women to disobey the recommendations or advices from health care practitioners [22].

The taboos of the same food or food group vary based on differences in regions, culture, and beliefs or reasons. In Indonesia, papaya (*Carica papaya* L.) is encouraged during pregnancy as it is classified as “cold” food [20], but in India it is discouraged as it may lead to a miscarriage [23]. Again, eggs are believed to cause bald-headed babies, in Zambia [24], and cause pregnant woman to behave like a chicken during delivery, which may in turn extend delivery period in Indonesia [20]. Further, in Ghana, eggs are avoided because they cause overweight in the fetus, which contributes to difficulties during child birth and possible death of the mother [21].

The classification of foods as either “hot” or “cold” in some Asian countries has an impact on pregnant women because pregnancy is understood as a hot condition, and thus they avoid “hot” foods [25]. “Hot” foods that should be avoided include high protein food such as fish, lentils (*Lens culinaris* Medikus), meat, eggs, beans (*Phaseolus vulgaris* L.), and yoghurt [25–28]. The general belief is that “hot” foods are harmful and “cold” foods are beneficial during pregnancy. “Hot” foods are encouraged during the last stage of pregnancy to aid the expulsion of the foetus [29]. “Cold” foods such as cucumber (*Cucumis sativus* L.), watermelon (*Citrullus lanatus* (Thunb.) Matsum. & Nakai), young coconut (*Cocos nucifera* L.), squash (*Cucurbita* spp.), and papaya are recommended during pregnancy because they are believed to increase body water and enhance fetus comfortability in the womb [20]. The “hot” and “cold” theory of humoral medicine is deeply rooted in Asia, Mexico, Latin America, north America, Europe, and some African Countries [30]. This is a doctrine of the four humors, with the belief that the human body contains four substances or humors (blood, phlegm, black bile, and yellow bile) with paired qualities of heat, cold, moisture, and dryness. According to this medicinal system, illness is explained by imbalance in the humors, and subsequently, treatment is designed to restore equilibrium. Eating habits have been identified as one of the factors that place the body in a vulnerable situation [31]. The theory has different perception and interpretation which varies within the same and also among different cultures. In Ayurvedic and Chinese humoral medicine, foods, medicinal remedies and other substances are characterized by pairs of metaphoric values of qualities: hot or cold paired with wet or dry [32]. The human body is subject to metaphoric insults (hot or cold), primarily from food and drink, and to natural thermal effects (also hot and cold) from environmental exposure to sun, rain, and cold air, which cause illness by upsetting the normal body equilibrium. People hope to avoid illness by balancing food and drink intake and avoiding excessive exposure to the elements. When they failed in the effort and fall ill, therapy is based upon the “principle of opposite” a hot remedy for an illness caused by an excess of cold, and a cold remedy for illness caused by an excess of heat [33]. Thus, treatment focuses on restoring body equilibrium by observing correct diet [34]. Ethno-physiology emphasizes on a balance of hot and cold concept for well-being, particularly because pregnancy generates a state of hotness, and thus it is desirable to maintain body equilibrium by taking cold foods [26]. The concept of “hot” and “cold” food is not common in South Africa, particularly in the Zulu culture.

Several studies have revealed that several women also practice a special food diet during pregnancy and lactation [8, 35]. Consumption of special food is mainly to improve the quality and quantity of milk, strengthen the baby, and improve hemoglobin [5]. The World Health Organization (WHO) [36] recommend that a pregnant woman should consume foods that has adequate amount of folic acid, iodine, calcium, vitamin D, vitamin B_12_, vitamin B_6_, vitamin A, vitamin E, vitamin K, choline, copper, magnesium, sodium, and zinc in order to ensure successful pregnancy. Postpartum is a critical period worldwide because most maternal deaths occur during this period [37]. Recommendations for nutrients intake during this period are based on replenishing nutrient stores, specifically, calcium, vitamin B_6_, and folate as well as supporting requirements for lactation when the woman is breastfeeding [37]. Nutritional status of breastfeeding women is of vital importance and adequate nutrition begins during pregnancy, when reserves are accumulating [38].

Maternal and child health are inseparable, thus whatever affects the mother’s health, usually affects the child [39]. A number of traditional food taboos adopted by pregnant women are carried over to their children after delivery. These practices include not giving children certain foods [40]. Food taboos also have a strong effect on infant feeding and undermine optimal infant feeding practices [41]. The belief of restricting children from eating protein-rich foods such as meat and eggs, because it will cause children to steal as meat and eggs are normally stolen by dogs, deprive children of these nutritious foods, which may lead to malnutrition [40]. The World Health Organization [42] reported that 45% of global deaths among children under 5 years of age are attributed to under-nutrition, which is approximately three million deaths each year. Cultural customs, taboos, and beliefs contribute to some of the reasons for malnutrition according to UNICEF [43].

The World Health Organization [44] recommended that after 6 months of exclusive breastfeeding, appropriate and adequate complementary foods should be introduced. Optimal nutrition during first years of life is crucial for optimal growth and development and possibly prevention of chronic diseases in adulthood [45]. Inadequate feeding practice is often a greater factor of malnutrition than lack of food [46]. Nutrient deficiencies that occur in the first years of life may have a negative impact on brain growth [47]. A study done in a rural part of KwaZulu-Natal reported high prevalence of anemia (49%), vitamin A deficiency (20%), zinc deficiency (32%), and iron deficiency (35%) in infants aged 6–12 months [48]. Cultural practice is one of the barriers to optimal complementary feeding practices and need to be addressed through nutritional program mediation [49].

Food habits of people are variously influenced by their culture and occupation [50]. Some previous studies have indicated insignificant differences in the adherence of food taboos between people who have and those who have not received formal education [4, 51]. Again, among well-educated women, the relationship between education and food beliefs is insignificant [52]. In a study done by Zepro [12], education was found to be an important determinant for health, where educated women acknowledged the importance of balanced diet during pregnancy compared to those with no formal education.

Age of the mother has also been associated with food taboos, as a study in Ethiopia reported that elderly women adhere more to food taboos than younger women [14]. The studies on the relationship between income status and food taboos has indicated that people with more income have less observance of food taboos [53–55]. Religion can also play a role to either encourage or deter people from observing food taboos [15, 56]. Christians in Ethiopia including pregnant women are not permitted to consume animal-based foods during fasting [57]. Such food taboo has been documented to affect pregnant women and their newborn babies [11, 15, 56]. The similar taboo is also practiced among Zimbabwean Christians, where pregnant women are not allowed to consume food such as pork and eggs [58].

Very little knowledge on the food taboos and practices of Zulu women during pregnancy, postpartum period, and infant feeding is documented in the literature. It is thus important to evaluate the prevalence of food taboos and assess food that is taken during pregnancy, postpartum recovery, and infant feeding, and to establish the extent at which women in the rural communities adhere to these food taboos. Indigenous knowledge, attitudes, and practices toward child feeding in the study area is not previously documented. Thus this study is also assessing indigenous mother’s knowledge, attitude, and practice on child feeding under 2 years. Mediation programmes that provide nutritional advice can then address these health concerns and the assumptions that underlie successful pregnancy and delivery, given the deep-rooted nature of its cultural beliefs. To address above objectives, the following three questions needs to be answer during this survey. Firstly, what food categories and practices are involved in taboos for pregnant women and infants? Secondly, what factors are associated with adherence to food taboos during pregnancy, postpartum period, and infant care; and thirdly, what is the impact of food taboos to the nutritional status of pregnant and nursing women as well as infants? From these questions, the following hypotheses were formed: Firstly, fruits, vegetables, some staple foods, and various liquids are either promoted or restricted for consumption by pregnant women and infants. Secondly, diversity in age, beliefs, or religion and educational level can either enhance or inhibit adherence to food taboos that are practiced during pregnancy, postpartum period, and infant care. Thirdly, that most of the nutrient-rich foods that are restricted from consumption because of taboos, leads to malnutrition of pregnant and nursing women as well as infants.

## Material and methods

### Study area

The study was conducted in northern Maputaland which is located in the north-eastern part of KwaZulu-Natal, one of the nine provinces of South Africa. This region is dominated by isiZulu speaking people. Northern Maputaland (Umhlabuyalingana Municipality Zone) is an economically deprived area with approximately 44.9% of the population (total population of 163,694 people) not having a formal income [59]; only 3% of the population is receiving an income that is more than R1600 (96$ at 13 September 2020) per month. This is possible because only 2% of the population has obtained tertiary qualifications. Fifty-four percent of the households are headed by females. Households are made up of a young population with only 4% of the members older than 65 years. This region consists of two hospitals and seventeen clinics [59].

### Ethnobotanical data collection

The survey was conducted with women in rural areas of northern Maputaland. One hundred forty questionnaires were conducted between the period of 2017 and 2020. Ethical clearance was received from University of Zululand [UZREC 171110-030] prior the onset of the survey. Written permission of the community leaders was also obtained from the Mashabane Traditional Council. Responses to the structured questionnaires designed for this study were collected by means of one-on-one interviews, where interviewed participants were chosen purposively, based on the women’s experience in pregnancy, postpartum recovery, and early child care and willingness to share the knowledge with the researchers. The objective of the study was explained in their native language (isiZulu) and consent form was signed prior to participation. The consent form clearly stated that their responses will be anonymous and they do not need to respond to any question they are not comfortable with. Again, the interviewer was a woman of the same culture as the interviewees, who clearly understood the emotional consequences of the questions asked. Also, being a mother herself, she knew how to handle such questions with empathy. Homesteads location was recorded using the global positioning system (GPS). This study was limited to pregnant women and mothers. Thirty-seven interviews were conducted in three schools (Emlambongwenya Primary School, Ingwavuma High School, and Nyamane High School). Fifteen interviews were conducted with farm workers at Coastal Cashew Farm, whereas the remaining interviews (88) were conducted with lay persons in their homesteads (Table 1).

**Table 1:** Employment/engagement type of the participants

Employment/engagement type of the participants	Number of participants
Farm workers	15
Educators (school teachers)	24
Laboratory assistants	2
Lecturers at a tertiary institute	2
Learners (grade 12)	2
Learner support agents	2
School cleaners	4
School cookers	5
Self-employed	13
Unemployed	71

Structured questionnaires were used to obtain information including socio-demographic information such as age, religion, educational background, occupation, number of pregnancies, and number of children alive, number of children that died before the age of two, place of giving birth, as well as their attendance of antenatal clinics. The questionnaire included questions on food avoided during pregnancy, food recommended during pregnancy, food/beverages that are taken to encourage lactation and postpartum recovery, as well as the food groups that are restricted for young children (2 years and younger). The reasons behind all the food taboos and cultural beliefs were also documented. Participants were also asked to give reasons for their actions and non-actions. Additionally, respondents were asked to ascertain whether or not they followed these traditional beliefs and the reason behind following the tradition. Data were analyzed using descriptive statistics.

## Results

### Socio-demographic information

In a total of 140 interviewed women with an age range from 18 to 90 years, the highest percentage (48%) of participants was from the middle aged (31–50 years) group followed by an older aged (51–90 years) group of 31%, and the smallest percentage (21%) was from the youngest aged (18–30 years) group. Education levels varied widely among participants, with 84% having received some level of formal education but the remaining 16% did not have formal education. Among those who had formal education, some had attended primary school (18%), others had proceeded to secondary school (45%), and a few had obtained tertiary qualifications (21%). Employment levels also varied, where more than half of the participants (52%) were unemployed; 9% were self-employed; and just 39% were employed (Table 1). All participants had experienced pregnancy and reported a total of 511 pregnancies. The majority (86%) of participants gave birth in clinics and hospitals, whereas only 14% delivered their babies at home, with assistance from parents, domestic workers, or elderly people in the community, and two of the participants had themselves assisted with deliveries.

Of the children born to the participants, as many as 34 (7%) were either stillborn or had died before the age of 2 years. Contributing factors of these deaths may include improper medical procedures as 53% of deaths reported occurred on pregnancies that were delivered at home. Among the older women (between 56 and 90 years)], 4% claimed that they had never attended antenatal clinics during any of their pregnancies because, at that time, there had been a limited number of clinics in the area. One of them reported having used traditional medicine to maintain a healthy pregnancy. Among those who attended antenatal classes, the stage at which they started to attend varied dramatically, from the second to the eighth month of pregnancy. Ninety-one percent of the participants were Christians. Majority of them believed in tradition and they were following these traditional food taboos and practices. However, the minority regarded food taboos and cultural beliefs as ancestral practices which are contrary to Christianity.

### Food avoidance during pregnancy

Table 2 shows food avoided by pregnant women from northern Maputaland. Some foods such as chili (*Capsicum* spp.), eggs, fruits [orange (*Citrus sinensis* (L.) Osbeck), mango (*Mangifera indica* L.), papaya, naartjie (*Citrus reticulata* Blanco), peach (*Prunus persica* (L.) Batsch)], and butternut (*Cucurbita moschata* Duchesne ex Poir.) were avoided because they were believed to affect the health of the fetus, where the majority reasoned these foods as affecting the skin of the unborn baby; followed by the birth of a baby with no hair; and they may cause jaundice. Foods such as beans and tomatoes (*Solanum lycopersicum* L.) are known to affect mothers instead of fetus and are avoided because they are believed to cause heartburn in pregnant women. Although rice (*Oryza sativa* L.) does not affect the health of the fetus or the mother, it was also avoided during pregnancy because they believe that it does not have nutritional value for the baby.

**Table 2:** Reasons for avoiding certain foods and liquids during pregnancy

Education background						
None *n* = 23	Grades 1–7 *n* = 24	Grades 8–12 *n* = 63	TEd *n* = 30	Total *n* = 140 (%)	Food and liquid avoided	Reason(s)
17	14	46	25	**102 (73)**	Butternut (*Cucurbita moschata* Duchesne ex Poir.), mango (*Mangifera indica* L*.*), Naartjie (*Citrus reticulata* Blanco*)*, papaya *(Carica papaya* L.) , orange (*Citrus sinensis* (L.) Osbeck), peach (*Prunus persica* (L.) Batsch)	Baby will have jaundice, hair loss
11	18	42	15	**86 (61)**	Chili (*Capsicum* spp.), ice	Baby will have burned skin or dark marks, rash and blisters appear before birth, causes diarrhea, causes red spot (*ibala*), the baby will cry a lot
12	13	38	16	**79 (56)**	Eggs	Baby will have no hair, causes jaundice
6	7	34	11	**58 (41)**	Sweets, sugarcane (*Saccharum officinarum* L.), commercial fruit Juice, sweet food, honey	Baby will drool after birth, have too much saliva, and develop eczema
7	9	23	10	**49 (35)**	Alcohol	Baby will be born sick, unhealthy, disabled or brain damage
6	6	11	6	**29 (21)**	I*mifino* (green leafy vegetables)	Baby will have too much saliva after birth, burn the skin of the baby, cause dark marks on skin
7	4	12	5	**28 (20)**	Amasi (fermented milk) or Milk	Baby will vomit and have heartburn after birth, too much dandruff, baby will be born with white stuff on the head, the baby will be lazy inside and not being clean when born
3	3	13	4	**23 (16)**	Any cold food, drink and ice	Baby will get pneumonia, cause kidney problems, may cause the baby to have skin problem and be underweight
2	3	15	2	**22 (15)**	Coke, soft drink, fizzy drinks	Burns baby skin, reduce milk production, causes asthma, acid will harm the baby, causes high blood pressure to the mother
2	3	2	1	**8 (6)**	Too much fat (oily food)	Mother will develop high blood pressure, increases heart rate, the baby will get fat, causes heart disease to the baby
2		5	1	**8 (6)**	Caffeine energy drinks, coffee	Causes abortion or miscarriage, increases blood pressure
	2	4	2	**8 (6)**	Liver	Baby will be born with no hair
		1	3	**4 (3)**	Lemon (*Citrus limon* (L. Osbeck)	Will have a small (underweight baby)
	2	2		**4 (3)**	Tea (hot)	Burn skin of the baby
2			1	**3 (2)**	Red meat	The baby will have gout, allergies, causes skin irritation
1	1	1		**3 (2)**	Bird meat	Baby will be very small, will talk too much, mother will have low supply of milk
		2	1	**3 (2)**	Soil	Block the tubes, causes jaundice, the baby will come out holding the soil
	1	2		**3 (2)**	Peanuts (*Arachis hypogaea* L.)	Baby will come out with whitish stuff, baby will vomit a lot
1		1		**2 (1)**	Goat meat	If the father’s surname is Mathenjwa and you eat goat meat, the baby will be stupid or crazy, if the father’s surname is Dlamini and you eat goat meat, the baby will lose hearing ability
1			1	**2 (1)**	Sour food	Baby will have malnutrition or skinny
		2		**2 (1)**	Salt	Causes high blood pressure
		2		**2 (1)**	Beans (*Phaseolus vulgaris* L.)	Gives the mother heartburn
		2		**2 (1)**	*Muthi* (herbal mixture)	Harms the baby
1		1		**2 (1)**	Potatoes (*Solanum tuberosum* L.)	Increases the baby’s weight making it harder to deliver
		2		**2 (1)**	Pineapple (*Ananas comosus* (L.) Merr.)	Pineapple causes miscarriage in early pregnancy
1				**1 (0.7)**	Rabbit meat	Baby’s eyes will always be open like a rabbit
1				**1 (0.7)**	Rice (*Oryza sativa* L.)	No growth value for the baby
	1			**1 (0.7)**	Polony (process sausage)	Baby will have no hair
	1			**1 (0.7)**	Drinking too much water	Baby’s joints will be weak
1				**1 (0.7)**	Bone marrow	Baby will have runny nose
	1			**1 (0.7)**	*Intethe* (grasshopper) (*Caelifera* spp*.*)	Baby will have a long head
		1		**1 (0.7)**	Butternut and pumpkin (*Cucurbita pepo* L.)	Baby’s face will be dark black
		1		**1 (0.7)**	Apples (*Malus domestica* Borkh)	The baby will be born bald
		1		**1 (0.7)**	*Mganu* alcohol (Marula beer) *(Sclerocarya birrea (A.Rich) Hochst.subsp.caffra (Sond.)Kokwaro)*	The baby will become a drunkard
			1	**1 (0.7)**	Laxatives	Induces abortion
			1	**1 (0.7)**	Chicken feet	Baby will have 6 fingers
			1	**1 (0.7)**	Samp (crushed white maize)	Baby will be skinny
			1	**1 (0.7)**	Sea food	Baby will be born bald
			1	**1 (0.7)**	*Isithubi* (made from cow milk)	Because you are pregnant, no specific reason
			1	**1 (0.7)**	Tomatoes (*Solanum lycopersicum* L.)	Causes heartburn to mother
		1		**1 (0.7)**	A*mabele* (*Sorghum bicolor* (L.) Moench)	Causes sexual activeness
1				**1 (0.7)**	Meat prepared for ceremonies	Considered as unhealthy
			1	**1 (0.7)**	Fruits	Causes excess body weight on the baby
			1	**1 (0.7)**	*Amadumbe* leaves (*Colocasia esculenta* (L.) Schott)	Affects the eyesight of the baby
			1	**1 (0.7)**	*Imbati* (*Obetia tenax* (N.E.Br.) Friis)	Affects the eyesight of the baby

*n* number of participants responded, *TEd* tertiary education, *Grade* refers to the twelve classes (grades 1–12) that one attends at basic education before qualifying for the senior certificate to tertiary institutions

Food avoidance is also based on health reasons particularly the fear of high blood pressure. Some women (6%) perceive that foods that are rich in fats and salts can cause high blood pressure. Other health reasons of avoiding these food groups is that the baby will get fat and the consumption of these food may cause heart disease to the unborn baby. Food taboos according to family clans including surnames such as Mathenjwa and Dlamini were also documented. One of the participants reported that if a pregnant woman is carrying a baby from the Mathenjwa clan she is not supposed to eat goat meat because the baby will either be stupid or mentally disturbed. Food such as *amabele* (*Sorghum bicolor* (L.) Moench), sea food, chicken feet, apples (*Malus domestica* Borkh), and rice were mentioned as food taboos during pregnancy but were only cited by less than 1% of the participants. One of the interesting taboos mentioned was avoidance of eating chicken feet because the baby will be born with six fingers.

### Food recommended during pregnancy

Leafy vegetables were mentioned by 51% of the participants as the most recommended food consumed during pregnancy (Table 3). Most mentioned reasons for recommending particular food was that they result in a healthy baby, provide vitamins, as well as increasing and cleaning the blood. Some of the foods were recommended because they give the baby certain features. These include consumption of fish, which makes the baby to be clever, whereas apples are believed to lead into having a baby with white eyes. Some of the foods were recommended because they also help to ease labor. Consumption of *Corchorus olitorius* L.“*igushe”* (slimy leafy vegetable) leaves was known to help dilate the uterus during delivery. Raw or boiled eggs were also recommended to facilitate labor. One of interesting drinks recommended during pregnancy was traditional beer commonly known as *ijuba*, which was believed to makes the baby to be pretty and lighter in skin color.

**Table 3:** Food and liquid recommended during pregnancy and the reasons

Education background						
None *n* = 23	Grades 1–7 *n* = 24	Grades 8–12 *n* = 63	TEd *n* = 30	Total *n* = 140 (%)	Recommended food and liquid	Reason(s)
11	13	33	15	**72 (51)**	Vegetables (leafy)	Provides vitamins, increases blood, healthy mind, builds bones, improves growth of the baby
4	5	15	12	**36 (26)**	Fruits	Healthy for baby
2	1	12	6	**21 (15)**	Liver	Restores, increases and cleanses blood
1	1	6	6	**14 (10)**	Fish	Makes the baby clever, delivery will be easy like a fish swimming in water, restores blood
4	1	6	2	**12 (9)**	Peanuts	Healthy for the baby, protect one’s body from infections, a good source of healthy oil
2	3	5	2	**12 (9)**	Phuthu pap (maize porridge) and Samp	Healthy for baby, energy for the baby
	2	4	6	**12 (9)**	Milk	For health and lactation reasons, strong bones, baby will be light in complexion, strong teeth, white eyes
4	4	2	1	**11 (8)**	Red meat, meat	Provide vitamins
4	1	1	4	**10 (7)**	Amasi (sour milk)	Provides good nutrition for the baby/lightens the skin of baby
	1	4	5	**10 (7)**	Water	Avoid dehydrating and for healthy baby
1	3	4	1	**9 (6)**	Apple	Give white eyes (cleans the eyes), strengthens the babies bones and makes the baby’s skin to be fresh
1	4	3		**8 (6)**	Beans	Healthy for the baby, strong bones
	1	4	2	**7 (5)**	Beetroot (*Beta vulgaris* L.)	Increases and cleanses blood
	1	5		**6 (4)**	Carrot (*Daucus carota* (Hoffm.) Schubl. & G.Martens)	Clear the eyes of a baby and the mother, good eye sight, promote brain development of the baby
		1	3	**4 (3)**	*Igushe* (slimy leafy vegetable) (*Corchorus olitorius* L.)	To helps dilate the uterus when one deliver
		1	2	**3 (2)**	Banana (*Musa acuminata* Colla)	Gives mother strength/for nutrients, help monitor urination if one is traveling a long distance
1		2		**3 (2)**	Yogurt	For Healthy baby with fresh and beautiful skin
		2	1	**3 (2)**	*Ijuba* (traditional beer)	Makes the baby pretty and lighter in skin color
			2	**2 (1)**	Prickly pear fruit (*Opuntia* spp.)	Because it is a slippery fruit it will ease the delivery, baby will be comfortable inside you
		2		**2 (1)**	Raw egg, boiled egg	To facilitates labor
		1	1	**2 (1)**	Cheese	Healthy for baby, causes the baby’s skin to be fresh
	1			**1 (0.7)**	*Ihlala* fruit (*Strychnos spinosa* Lam.)	Healthy for baby
1				**1 (0.7)**	Chicken	Healthy for baby
1				**1 (0.7)**	Maize (*Zea mays* L.)	Healthy for baby
1				**1 (0.7)**	Brown bread	Healthy for the baby
		1		**1 (0.7)**	Starchy food	Improve fitness of the baby
		1		**1 (0.7)**	Tomato	Clean the baby’s eyes
		1		**1 (0.7)**	Mopane worms (*Gonimbrasia belina* Westwood)	Provides Protein for the baby
			1	**1 (0.7)**	Rooibos tea (*Aspalanthus linearis* (Burm.f.) R. Dahlgren)	Cleans the system of the baby
			1	**1 (0.7)**	*Amadumbe*	Healthy for baby

*n* number of participants responded, *TEd* tertiary education, *Grade* refers to the twelve classes (grades 1–12) that one attends at basic education before qualifying for the senior certificate to tertiary institutions

### Specific food taken (recommended) during the postpartum period (for recovery and encouraging lactation)

Soft maize porridge was the most recommended food to be consumed after delivery to regain strength and facilitate lactation (Table 4). The most cited reasons for consumption of these foods were that they restore strength, encourage lactation, and increase blood. Latex from a plant called *Ingontshwa*/*amabhelebhele* [*Sarcostemma viminale* (L) R. Br.] was also cited to be taken to facilitate lactation. The plant was identified by its isiZulu name, and was used in the same area where the research was conducted.

**Table 4:** Specific food taken during the postpartum period by the mother and the reasons for it

Education background						
None *n* = 23	Grades 1–7 *n* = 24	Grades 8–12 *n* = 63	TEd *n* = 30	Total *n* = 140 (%)	Food taken during postpartum	Reason
13	16	44	8	**81 (58)**	Soft porridge (maize porridge)	Restore strength and encourage lactation
5	8	15	9	**37 (26)**	All fruits and vegetables	Increases and restores blood
6	4	15	11	**36 (26)**	Beetroot	To cleanse and restore loss blood and to avoid constipation
4	7	15	2	**28 (20)**	Tea	Encourages lactation
2		14	8	**24 (17)**	Liver	Increases blood
2	2	18	3	**25 (18)**	*Mageu* (fortified maize drink)	Encourages lactation, gives strength and energy
2	5	4	6	**17 (12)**	Meat	Build muscles
2	4	9	2	**17 (12)**	Amasi (fermented milk)	Restore strength and blood, facilitates lactation
2	4	8	1	**15 (11)**	Coffee, milo	Encourages lactation
		5	6	**11 (8)**	Spinach (*Spinacia oleracea* L.) and *Mfino* (leafy vegetables)	Healthy, provides energy, Increases lactation, restores blood
1	1	4	3	**9 (6)**	Samp (crushed maize)	Restores energy
1		4	1	**6 (40**	Beans	Restores energy, boosts lactation
2		2	1	5 (4)	Peanuts	Restore strength and blood
	1	2	1	**4 (3)**	Eggs	Healthy, increases the blood
1	1		1	**3 (2)**	Hot porridge	Burns the dirty blood after delivery, to regain energy
		2	1	**3 (2)**	Yoghurt	If you have a cesarean delivery, it prevents a big tummy
		2	1	**3 (2)**	Water	Facilitates lactation
		3		**3 (2)**	Pumpkin	Restores energy
		2		**2 (1)**	Cheese, polony (processed sausage)	Provide vitamins
1				**1 (0.7)**	Maize bread	Restores energy and builds muscles
			1	**1 (0.7)**	Chicken soup	Warms the body after delivery
			1	**1 (0.7)**	Brown bread	Restores energy
1		1		**1 (0.7)**	*Ingontshwa/amabhelebhele* plant (latex) (*Sarcostemma viminale* (L) R. Br)	Encourages lactation
	1			**1 (0.7)**	*Isijingi* (porridge made from *Citrullus lanatus* (Thunb.) Matsum. & Nakai)	Facilitates lactation
		1		**1 (0.7)**	Juice	Facilitates lactation
		1		**1 (0.7)**	*Amadumbe*	Restores energy
		1		**1 (0.7)**	Coke	Facilitates lactation
		1		**1 (0.7)**	Mayonnaise	To regain strength
		1		**1 (0.7)**	Fish	To regain blood

*n* number of participants responded, *TEd* tertiary education, *Grade* refers to the twelve classes (grades 1–12) that one attends at basic education before qualifying for the senior certificate to tertiary institutions

### Food restrictions to children below 2 years

Meat was the well-known food that is restricted from children below 2 years (Table 5). Most cited reasons for restricting certain foods was that children’s teeth/digestive system is not strong, causes rotten teeth, and may result in malnutrition. One of the interesting foods avoided was salty foods as it is believed that salt goes to the knees of the baby and can delay the walking process and can also delay the development of their teeth.

**Table 5:** Food that should not be given to children below two years and reasons for it

Education background						
None *n* = 23	Grades 1–7 *n* = 24	Grades 8–12 *n* = 63	TEd *n* = 30	Total *n* = 140 (%)	Food avoidance for child < 2 years	Reason (s)
10	8	11	10	**39 (28)**	Meat	Teeth and digestive system are not strong, the child will craves meat his whole life or craves other kids food, the baby will grow up sexually active, causes diarrhea and intestinal worms
3	7	22	3	**35 (25)**	Sugar and sweets	Rotten teeth, too much saliva and sores on tongue
7	3	12	1	**23 (16)**	Strong food (cereal, *amadumbe*, dry corn, sweet potatoes (*Ipomoea batatas* (L.) lam)	Hard to digest due the child’s underdeveloped digestive system
1	2	7	2	**12 (9)**	Fizzy drinks	Causes asthma
1	2	2	5	**10 (7)**	Water	The water will go to the knees and delays walking process, disturbed the stomach
1	2	6	1	**10 (7)**	Banana, sweet potato	Constipation, causes diarrhea, rotten teeth
1	4	1	3	**9 (6)**	Samp and beans	Teeth are not strong, cannot digest it
2	3	4		**9 (6)**	Salty food	The salt will goes to the knees and delays walking process/the process of having teeth will take longer
3	1	3	1	**8 (6)**	Ox liver	Will poop inside house for the rest of his/her life, very active bile, the baby will be lazy, the baby will be stupid, does not digest well
2	2	3		**7 (5)**	Amasi milk (fermented milk)	Causes tape worms and diarrhea
	2	4	1	**7 (5)**	Yoghurt and purity	Baby will not grow well, cause fever and intestinal worms.
	1	3	3	**7 (5)**	Milk, cheese, chicken and polony (process sausage)	They speed up maturation
		4	2	**6 (4)**	Phuthu pap (maize porridge)	Causes intestinal worms, slow digestion, causes kwashiorkor
		5		**5 (4)**	Chili, spices	Ulcers, runny tummy, can also irritate the skin.
		1	4	**5 (4)**	Eggs	Speeds maturation process
	3	1		**4 (3)**	Cakes, cookies	Cause teeth decay
	1	2		**3 (2)**	Hot potato chips	Skin problems/cause ring worms
		1	2	**3 (2)**	Soft porridge which is not supplemented by milk	The baby will have kwashiorkor (malnutrition)
1		2		**3 (2)**	Tea	Causes headache and can be addictive
1		1		**2 (1)**	Alcohol	Brain damage
		1		**1 (0.7)**	Chicken to a girl	The child will lust over men and be sexually active at an early stage
			2	**2 (1)**	Cabbage (*Brassica oleracea* L.)	Diarrhea/causes constipation
		1		**1 (0.7)**	Food related to the child’s surname is taboo	Child will get sick
		1		**1 (0.7)**	Honey	Baby will suffocate because it is too thick
		1		**1 (0.7)**	Fish	Fish contain bones that may harm the baby

*n* number of participants responded, *TEd* tertiary education, *Grade* refers to the twelve classes (grades 1–12) that one attends at basic education before qualifying for the senior certificate to tertiary institutions

### Traditional food practice adherence by the participants

Sixty-four percent of the participants in the current study were adhering to traditional food practices during pregnancy, postpartum period, and infant feeding. Participants believe that miscarriage or still born and some health complications such as jaundice, eczema, and malnutrition in new born babies are linked to consumption of restricted food, with two percent of the participants claiming that they had a personal experience of such. Thirty-six percent of the participants reported that although they know traditional beliefs, they are not following these taboos. The reasons ranged from believing that traditional beliefs do not work (14%); due to food cravings (16%); prohibited by their religion (Christianity) to believe in any traditional things hence they believe in information they receive in clinics (3%); and because their families did not teach them about traditions (3%).

Our results showed no significant difference in educational level and income status of the women who followed food taboos and those that did not follow them. Among the sixty-four percent of women who were following these traditional food practices, they claimed to do so for various reasons. The majority of women (46%) claimed to follow these traditional food practices for the baby’s health followed by those who were adhering for their own health (21%). Other reasons for adherence to the traditional food practices included that participants valued their families’ opinion (11%), cultural respect (9%), for easy delivery (9%), respect of the ancestors (2%), as well as the respect for the community (2%).

## Discussion

This study showed that food taboos still exist alongside with the use of modern health care services. Sixty-four percent of the participants in the current study are still practicing food taboos and following traditional recommendations during pregnancy, postpartum period, and infant feeding. This high level of practice accords with results of other studies that were conducted in Asian countries including Indonesia [20], Thailand [60], China [61], India [8], Cambodia [62], central Asia [63], as well as in African countries including Ghana [21], Ethiopia [11], Nigeria [13], Kenya [30], Sudan [18], and South Africa [64, 65]. Our study differs from previous studies conducted in South Africa because it focused on the Zulu culture of women living in a rural area. The study did not only focus on dietary practices during pregnancy and postpartum recovery, but it also extended to dietary practice during infant feeding.

The majority (96%) of women interviewed in the current study were attending antenatal classes. Health care workers emphasize preservation of pregnancy by eating a well-balanced diet. During antenatal classes, emphasis is given to a diet high in protein, vegetables and fruits, while avoiding tobacco and alcohol, and wearing loose clothes to enhance comfort [66]. Several studies have evaluated the nutritional status of pregnant women and infants in South Africa [48, 67–70]. A study by Chakona and Shackleton [65] highlighted that cultural beliefs is one of the reasons for women to avoid nutritious foods during pregnancy. No study could be found in the literature that has evaluated reasons that lead to inability of health care workers to get the message through to pregnant women about nutritional requirements during pregnancy.

The higher number of participant within middle (31–50 years) and elderly (51–90 years) age groups in this study resulted from these age groups having the relevant information needed, and were the ones who were most easily available for interviews. Older women also had better knowledge than the young and middle age women on cultural food practices during pregnancy, postpartum period, and infant feeding, perhaps because they were culturally more experienced. Middle age and elderly women (40 and above) were interviewed in order to compare the information that the younger generation and older generation holds. Previous ethnobotanical surveys done in the same region indicated that indigenous knowledge is transferred from one generation to the other. The results indicated that older women hold more knowledge than the younger generation. This survey confirms that there is a loss of indigenous knowledge as each generation passes.

More than half of the participants (51%) in the current study were unemployed. It cannot be disputed that good nutrition is a major determinant of positive pregnancy outcome. The link between poverty and malnutrition during pregnancy has been well established in South Africa [69, 71], hence the study focusing on other contributing factors of malnutrition (such as food taboos) has been poorly established. Not much is known on the negative or positive effects of food taboos on pregnancy outcomes of Zulu women. A study done on Xhosa speaking people in the Eastern Cape, South Africa, also highlighted that cultural beliefs are some of major drivers which influence the eating habits during pregnancy and infant feeding [71]. It was thus important to evaluate if cultural beliefs also influence dietary intake of pregnant women in other cultures. Our results indicated that there is no difference in food avoided or consumed by participants who are employed and those that are not employed. Therefore, poverty is not a major contributing factor toward dietary intake during pregnancy in relation to cultural food taboos.

There were insignificant differences in educational level and income status of the women who followed food traditions and those that did not. Similarly, in Pakistan [51] and India [4], those who have received formal education and those who have not received formal education shared the same traditional misconceptions of dietary practice during pregnancy and postpartum period. In contrast, a study in Ethiopia reported that the value of a modern balanced diet during pregnancy was significantly associated with younger, more educated mothers who had attended antenatal clinics [12]. The analysis of the findings of the current study was structured thematically, with the findings, how it relates to literature and how the findings relate to modern science or medicine.

### Dietary restrictions during pregnancy

Our findings (Table 1) revealed that cultural food taboos meant that women would avoid certain foods that are essential for a healthy pregnancy. This causes concern, as almost all (96%) of our participant sample had attended antenatal clinics and would have been informed about dietary requirements during pregnancy. The women cited common and nutritious foods—including oranges, mangoes, naartjies, papayas, peaches, butternuts, and eggs—as being traditionally restricted. Yellow and orange fruits and vegetables were to be avoided, they explained, because of a belief that the baby would then be born with jaundice, a condition causing the newborn’s skin and eyes to become yellow. The same restriction on the grounds that such fruits were responsible for skin discoloration in the baby were also reported in a study in the Eastern Cape, South Africa [65]. The belief could be associated with doctrine of signature, which links yellow and orange fruits to yellow skin. Our study shows that both Zulu and Xhosa (Eastern Cape) cultures share this restriction and for the same reasons.

In previous studies, common reasons for restricting certain fruits during pregnancy included the fear of abortion or miscarriage. For example, in Asian countries, papaya is considered a “hot food” whose consumption could lead to miscarriage [4, 5]. Fruits traditionally to be avoided, as reported in our study, are in fact rich in vitamins A and/or C, both of which are essential during pregnancy. Vitamin A is important for cell division, fetal organ and skeletal growth and maturation [72], and its deficiency affects some 19 million pregnant women, mostly in Africa and south-east Asia [1]. Severe vitamin A deficiency in the mother can lead to low vitamin A reserves in the body, which can detrimentally affect the lung development and survival of the baby in the first year of life [73]. Vitamin C is an antioxidant required for the synthesis of collagen and for prevention of pre-eclamptic toxemia (characterized by high blood pressure, swollen ankles and protein in the urine) [36]. Dietary intake of vitamin C can be useful in sustaining pregnancy to term; its lack was found to cause premature rupture of the chorioamniotic membrane [74]. Vitamin C is also important for raising the uptake of iron [36]. Thus avoidance of these fruits can lead to complications for the unborn baby.

The current study reported traditional avoidance of eggs in pregnancy for Zulu women, due to a belief that the baby would be born with no hair. Previous studies have noted egg restrictions in various cultures during pregnancy, but for different reasons. They were taboo in Pendhalungan society (Indonesia), which believed that the baby would be born smelling fishy [75]. In India (Tumkur), eggs are restricted because they are thought to cause bluish discoloration in the baby. In Kenya (Uasin Gishu County), consumption of eggs in pregnancy is believed to make a fetus grow excessively big, which causes problems for the mother during childbirth [35]. In the Eastern Cape (South Africa), eggs are taboo for Xhosa people because they are believed to increase the mother’s sexual appetite, which can be shifted to the unborn female child [65].

However, science reveals that eggs offer good value for pregnant women at an affordable price, as they provide essential fatty acids, proteins, choline, vitamins A and B_12_, selenium, iodine, and critical nutrients at levels above or compared to those found in other animal-source food [76]. Consumption of eggs during pregnancy has the potential to improve the child’s birth outcomes and brain development. Inadequate intake of choline by the mother during pregnancy has been associated with neural tube defects and changes in brain structure and functions in the offspring [77]. The vitamin D in eggs can also prevent rickets in the newborn baby and keep the mother’s teeth and bones healthy [1]. Iodine deficiency can increase the risk of spontaneous abortion, perinatal mortality, birth defects, and neurological disorder [78]. Restricting egg consumption during pregnancy is therefore a concern, especially as it is common in many cultures.

*Salmonella* has been identified to cause several foodborne outbreaks, particularly where raw or minimally cooked eggs are consumed [79]. However, Duguid and North [80] indicated that freshly cooked eggs are relatively safe, as their bacterial load is usually too low to cause any infections. On the other hand, caution should be taken to give under-cooked eggs to pregnant women, children as well as elderly and sick people, as a low bacterial load can affect them [81]. Thus pregnant women will be advised to consume thoroughly cooked eggs to meet their daily protein requirements as eggs are cheaper than meat.

Almost two-thirds (61%) of our participants reported the belief that avoiding chili and ice during pregnancy could prevent the baby’s skin having burns or dark marks or rash or blisters before birth, diarrhea in the mother, red spot (*ibala*) on the back of the baby’s head, and excessive crying. Similar traditional restrictions were reported in Indonesia, where chili was avoided because it makes infants cry easily and also makes them dirty and sick [9]. However, no scientific evidence has been found of any side effects of the consumption of chili during pregnancy. On the contrary, they are a great source of vitamins A, C, and E [82]. They also have medicinal properties that include boosting immunity [83], treating diabetes and obesity [84], lowering blood pressure, and reducing heart attacks [85].

In Indonesia (Madura island), consuming ice/cold water was reported to be taboo as it makes the mother’s womb fertile, resulting in a large baby, which could complicate the birth process. In addition, it could cause the mother to give birth to conjoined twins or to experience bleeding during delivery [9]. The consumption of ice is pagophagia, commonly known as pica (the craving for and chewing of substances with no nutritional value), and is closely related to iron deficiency [86]. Women who report cravings for ice during pregnancy, therefore, could be reporting a symptom of a condition that needs to be addressed, and that could go undetected if the tradition of avoiding ice in pregnancy is enforced.

Some of the traditional Zulu food taboos reported in our study are beneficial to health and should be reinforced. Consumption of sweets, sugarcane (*Saccharum officinarum* L.), commercial juice, sweet food, and honey, for example, was also considered taboo during pregnancy as it could result in a drooling baby with a great deal of saliva and also cause eczema. In Laos in the People Democratic Republic, sugarcane was taboo because it would lead to a fat baby and thus a difficult delivery [87]. Avoidance of sugary foods in pregnancy was also reported in Kenya (Uasin Gishu County), where 11% of the participants believed that the sugar would make the baby salivate excessively. Sugary foods eaten by the mother could also give the baby colic and give the mother and baby malaria [35]. Health care workers support some of these restrictions. Sugar consumed during pregnancy can affect both mother and unborn child: it can shape feeding behaviors and taste preference in the offspring, and increase future possibility of obesity and related metabolic diseases [88]. High maternal intake of sugar during pregnancy can also increase the risk of atopic asthma in the baby [89]. Again, high consumption of added sugar during pregnancy has also been associated with the development of gestational diabetes mellitus [90]. Thus this is a worthy taboo as it prevents a variety of complications in a new-born.

Food taboos can protect mother and fetus from food toxin. For example, alcohol consumption was reported in the present study as a taboo for pregnant women, because it was believed to cause the baby to be born sick, unhealthy, disabled, or brain damaged. In Kenya, alcohol consumption in pregnancy is also taboo, because it is believed to suck the baby’s blood, thereby causing low birth weight and stunted children [35]. Science has shown alcohol to be a teratogen that can readily cross the placenta and result in irreversible damage to the brain and other organs of the developing fetus [91]. Prenatal alcohol exposure can cause fetal alcohol spectrum disorders, which include learning difficulties, executive dysfunction, impaired speech, motor problems, and behavioral issues [92]. Consumption of alcohol during pregnancy can also induce preterm labor, lead to decrease breast milk production and, in the first trimester, increase the risk of spontaneous miscarriage [93]. This traditional food taboo, therefore, protects women and their babies from side effects, and is consistent with the advice they receive from health care workers.

### Traditional food recommendations during pregnancy

Foods taken by the participants in the current study does not provide all essential nutrients required for successful pregnancy. Dairy, meat, eggs, nuts, and legumes were less recommended, thus this may lead to insufficient intake of some nutrients required. In the current study, 51% of the participants recommended leafy vegetables to be consumed when one is pregnant as they believed it provides vitamins, increases blood, healthy mind, build bones, and improve growth of the baby. However, some women (21%) perceived that eating green leafy vegetables is a taboo as they believe that the baby will be born with excessive saliva, burned skin or the baby will have dark marks on the skin. Thus, the consumptions of leafy vegetables during pregnancy is controversial as some participants believe it’s a taboo while others recommend it. In India (Tamilnadu), green leafy vegetables are considered as “cold food” and consumption of these food items during pregnancy causes a cold and fever to the mother [8]. Consumption of vegetables was recommended in Thailand, where some women claimed to consume them throughout the pregnancy and other only consume them toward the end of pregnancy [60]. According to a study done in Kenya, 89% of the participants recommended traditional leafy vegetables, as they are believed to increase the volume of the blood in woman’s body and build the body which gives women strength during labor [35].

Leafy vegetables are rich in iron and folate. Iron form the red blood cells for the mother and the baby. It helps to carry oxygen in the blood from the lungs to the tissue. The baby’s brain and body need iron and oxygen to grow [1]. Iron deficiency may cause anemia and inadequate intake of iron during pregnancy is associated with increased cardiovascular risk for the offspring in adult [94]. Folate plays an important role in many metabolic reactions such as biosynthesis of DNA and RNA, methylation of homocysteine to methionine, and amino acid metabolism. Inadequate dietary intake can lead to anemia, leucopenia, and thrombocytopenia [95]. Pregnant women should be given advises on the nutritional facts of leafy vegetable as some women are avoiding them because they believe that they may have some side effects on the unborn baby. There is no scientific evidence that support that leafy vegetables can cause skin problems to the unborn baby as mentioned by some participants.

The second most recommended food by the participants during pregnancy was fruits which are believed to result in having a healthy baby. Fruits recommended included all variety of fruits except those that were restricted [mango, naartjie, orange, papaya, peach]. In Indonesia, fruits were suggested during pregnancy because they do not cause nausea [9]. These results are similar to what was reported in Kenya, where 35% of the participants reported that eating fruits was recommended because fruits are believed to increases the mother’s blood volume. Fruits are also believed to improve the mother’s appetite; helping in digestion, and improve the skin (softness) of the baby after birth [35]. They are a rich source of vitamin A, vitamin C, and fiber. Fiber is required to prevent constipation, reduce the risk of gestational diabetes, and pre-eclampsia [36].

Liver was the third most recommended food during pregnancy in the current study. The women said it restores, increases, and cleanses blood. This result is similar to what was reported in Kenya, where 24% of the participants recommended liver during pregnancy as they believe that it increases the volume of the blood [36]. Liver is a good source of vitamins A, C, B_6_, iron, protein, and cobalamin. Vitamin A is important for visual health, immune function, and fetal growth and development [1]. It exists in two forms, preformed vitamin A which is found in animal sources as well as provitamin A (carotenoids) found in plant sources. Absorption of vitamin A from vegetable sources is poor, thus food of animal origin is necessary to achieve daily requirement [96]. Dietary intake of preformed vitamin A greater than 7000 μg may be teratogenic leading to increased risk of congenital malformations [97]. According to the WHO [36], pregnant women should avoid consumption of liver as it contains retinol thus women should be encouraged to consume more products of plant origin that contain carotenes (provitamins), which is not teratogenic to the fetus and less of preformed vitamin A.

Fish was also recommended during pregnancy as the participants believed that consumption of fish makes the baby clever, woman will deliver easy like a fish swimming in water and it also restore blood. The assumption of fish making the baby clever could be possible as fish consist of nutrients that are required for fetal neurodevelopment. In the study conducted in Tumkur (India), fish was recommended by 78% of the participants during pregnancy as it improves memory, IQ, and milk production [5]. However, in other studies, fish was considered a taboo, where in west Bengal (India), fish caught by a net are avoided because the net represent the uterus, thus, one will have to break the net in order to deliver the baby, which means delivery through cesarean section [10]. In another study done in Eastern Cape (South Africa), fish was considered a taboo because the baby will be born with scales and with a rash on the skin, eczema, or be born with no hair and rough skin with small pimples [65].

Fish is a rich source of protein, and other nutrients that are required for fetal neurodevelopment which include iodine, selenium, choline, vitamin D, iron, and long chain n-3 fatty acids [98]. Other health benefits of fish consumption during pregnancy include increased birth weight and reduced risk of spontaneous miscarriage [99]. Iodine is essential for the development of the fetal central nervous system [36]. Some fish also contains mercury (accumulated from polluted water) which cause acute toxicity in man and can pass through the placenta [100], thus it can affect the fetus and it is recommended that pregnant women minimize exposure to mercury [98]. Potential contamination of fish by mercury requires cautious consideration as a dietary recommendation of fish during pregnancy [101]. Research showed difficulty of balancing the benefits of fish with the risk of mercury intake [102]. Thus, women should be advised about the types of fish that are low in mercury and safe for consumption during pregnancy.

### Traditional food recommendations during postpartum recovery

The most recommended food in the current study was soft porridge (maize meal), as it is believed to restore strength and encourage lactation. Soft porridge is a bulk food of low-nutrient density, which is made by diluting maize meal (*Zea mays* L.) with water to obtain thin consistency [103]. Soft, not too strong, and not too spicy foods were also recommended during postpartum recovery in a study conducted in Brazil [104]. In Limpopo Province (South Africa), special meals such as warm soft porridge and indigenous vegetables with ground peanuts were reported to be consumed to encourage production of milk and promote recovery after birth [64]. Although soft porridge is easily prepared and digested, when consumed alone, it does not provide enough protein to boast lactation and restore energy. In order to regain strength and energy, breastfeeding mothers need a diet that is high in protein and carbohydrates for milk production [105], thus women need to be advised on dietary intake of protein to boast milk production.

The second most mentioned food taken during postpartum recovery in the current study was fruits and vegetables to increase and restore blood. These results are contradicting with a study done in China, where 18% of the participants never ate vegetables, while 78% never ate fruits during postpartum period [61]. “Cold foods” (vegetables) were restricted during postpartum recovery as it decreases breast milk production. Rather women were advised to consume “hot food” which includes fruits such as banana, coconut, pineapple and red chili as it helps the mother to recover from the trauma of labor [10]. In India, green leafy vegetables were not recommended during lactation because they are considered “cold foods” and can cause a cough and cold to the child [8].

Beetroot (*Beta vulgaris* L.) was also recommended for postpartum recovery to cleanse, restore loss blood, and to avoid constipation. The belief is that since beetroot is red in color, it symbolize blood, thus it has to be consumed after loss of blood during delivery. Beetroot is a good source of fiber, potassium, manganese, iron, vitamin C, folate, and other numerous vitamins and minerals [106]. Taking beetroot for 20 days was reported to increase the serum iron level, mild increase of hemoglobin and ferritin, thus beetroot might have some therapeutic properties for iron deficiency. It was suggested that beetroot should be put within the dietary protocols for women at childbearing age [107].

Women in the current study reported to consume high volumes of tea and coffee during postpartum recovery as they believe it encourage lactation. This also helps them to keep hydrated. However, they need to be advised about the types of tea to consume as some contain caffeine. The World Health Organization [44] recommended a limit intake of tea and coffee during pregnancy and lactation as it interferes with iron absorption. Caffeine can pass into the breast milk and cause hyperactivity and sleeping problems in the baby. Water was mentioned to be essential during postpartum period as it facilitate lactation. However, this contradicts a study done in India, where the participants reported that consumption of water is restricted as it may lead to the swelling of the stomach of the baby [5]. Water restriction was also mentioned in a study done in west Bengal [10].

### Food taboos in infants

The most common restricted food avoided in young children in the current study was meat. It was restricted because their teeth are not strong, they may crave meat their whole life or crave other children’s food, and the baby will grow up sexually active or it can cause diarrhea and worms. The current study differs from the study conducted in Laos in the People Democratic Republic, where young children were given everything the rest of the family eats [87]. In a study done in Mid-West state of Nigeria, meat and eggs were not given to children because of parents’ belief that it will cause children to steal as meat and eggs are normally stolen by dogs [108]. Animal products are the only foods that contain enough iron, zinc, calcium and riboflavin to supply daily requirements for complementary feeding, while being low in anti-nutrients [109].

The World Health Organization [44] recommended that meat, poultry, fish, and eggs should be eaten daily, or as often as possible. Meat provides an important role in a diet, as it provides nutrients which are amino acids, vitamin A, vitamin B_1_, vitamin B_2_, niacin, vitamin B_6_, vitamin B_12_, iron, and zinc [45]. Infants and children under the age of five years are at risk of developing iron deficiency anemia because of their increased requirements for rapid growth and diets that are often lacking sufficient absorbable iron [110]. Deficiency of iron in young children may lead to increased perinatal mortality, delayed mental and physical development, reduced auditory and visual function, and impaired physical performance [111].

The second most mentioned food avoided in the current study was sugar and sweets as they may cause rotten teeth, too much saliva and sores on the tongue. There is no nutritional requirement of any type of sugars in infants [112]. Thus these taboos align with medical nutritional requirements for infants. Added sugar contribute to poor health outcomes, which is a concern to children as excessive consumption of sugar has been linked to several abnormalities and adverse health conditions [113]. Sugar intake has been associated with increased risk of dental caries and adiposity (obesity) [112]. If parents feed their children food with sweet flavors, it is most likely to affect subsequent food preferences and eating behaviors [114]. Again, early-life exposure of excess sugar may also create a predisposition to non-communicable diseases [115]. Reducing added sugar intake and replacing it with water is associated with reduced weight and adiposity in children [112].

Food taboos during infancy and childhood are also intended to protect children during vulnerable stages in life [63]. Strong (chewable) foods such as cereal, *Amadumbe* (*Colocasia esculenta* (L.) Schott), dry corn, and sweet potatoes (*Ipomoea batatas* (L.) lam) were also avoided for young children because they are hard to digest due the child’s underdeveloped digestive system. In Tajikistan (Asia), heavy foods (bread, meat, vegetable filled pastry, rice dumpling filled with meat, and vegetables) were considered a taboo because it is difficult to digest these meals [63]. In some countries, people practice premastication, or chewing of food by the mother, father, grandmother, or sibling to soften strong foods [87]. This is done to ensure that infants with underdeveloped digestive system are able to get enough food to meet daily dietary requirements.

## Conclusions

The study revealed that food taboos and practices relating to pregnancy, postpartum, and infant feeding period do exist and a significant number of Zulu women in a rural area still practice them. Women adhere to these food taboos and practices for health and sociocultural reasons. The beliefs about the detrimental effects of some foods were not backed up by scientific research. Food taboos and practices still contribute to unhealthy nutrition in pregnancy and early childhood. While practicing food taboos and practices can expose women to poor nutrition, some food taboos can also potentially protect women against unhealthy eating. Thus, it is important to understand the dual impact of food taboos to develop effective, culturally sensitive, community-based programs. Although the majority of women were attending antenatal clinics, their food preferences during pregnancy, postpartum recovery, and early child care are more influenced by cultural beliefs. Thus, there is a need for nutrition education and awareness among pregnant women to reduce misconceptions. Programs should focus on changing the current knowledge, attitude, and practices. During pregnancy, not only deficiency but also excessive amounts of certain substances particularly vitamin A can be dangerous. Thus, the nutritional education should also emphasize the daily requirements of potential harmful nutrients. The study revealed that some of the food taboos and practice align with what is recommended by health care workers for a healthy pregnancy while others are contrary to the recommendations. The study indicated that infants are restricted from some food that are essential for growth, thus there is a need for mediation to improve nutritional related caregiving practices during the period of complementary feeding. Previous studies done in this rural area have documented that people normally use both traditional medicine and allopathic medicine. However, some of them prefer traditional medicine over allopathic medicine. Other ethnobotanical studies also confirmed the importance of traditional medicine in the primary health care within this rural area. It is thus not unusual for this community to still depend heavily on cultural food practices over the knowledge that they received from antenatal classes. Although the region has well established health care systems (two hospitals and seventeen clinics) which are easily accessible, women still prefer to practice the traditional cultural knowledge shared among the community.

## Data Availability

All data are included in the manuscript.

## References

[1] World Health Organization. Recommendations on antenatal care for a positive pregnancy experience. Geneva; 2016. Apps.who.int/iris/bitstream/handle/10665/250796/9789241549912eng.pdf.; jsessionid=1f1D3C626C011EB5F23C43745EE73DF?sequence=1 (Accessed 23/06/2020)

[2] World Health Organization. Healthy diet. Geneva; 2020. p. 9. Int/news-room/fact-sheets/detail/healthy-diet (Accessed 24/06/2020)

[3] Kominiarek MA, Rajan P (2016). Nutrition recommendations in pregnancy and lactation. Med Clin North Am.

[4] Patil R, Mittal A, Vendapriya DR, Khan MI, Raghavia M (2010). Taboos and misconceptions about food during pregnancy among rural population of Pondicherry. Calicut Med J.

[5] Shwetha TM, Shwetha R, Krishna I, Usha R (2017). Food taboos among pregnant and lactating mothers in Tumkur: a qualitative study. Int J Commun Med Public Health.

[6] Otoo P, Habib H, Ankomah A (2015). Food prohibitions and other traditional practices in pregnancy: a qualitative study in western region of Ghana. Adv Reprod Sci.

[7] Maduforo AN, Nwosu OIC, Ndiokwelu CI, Obiakor-Okeke PN (2013). Food superstition, feeding practices and nutritional anthropometry of pregnant women. Jorind.

[8] Banu KK, Prathipa A, Anandarajan B, Sheriff AMI, Muthukumar S, Selvakumar J (2016). Food taboos during antenatal and postpartum period among the women of rural and urban areas of Tamilnadu. Int J Biomed Res.

[9] Diana R, Rachmayanti RD, Anwar F, Khomsan A, Christianti F, Kusuma R. Food taboos and suggestions among Madurese pregnant women: a qualitative study. J Ethn Foods. 2018;5:246–53.

[10] Chakrabarti S, Chakrabarti A (2019). Food taboos in pregnancy and early lactation among women living in a rural area of west Bengal. J Family Med Prim Care.

[11] Desalegn K, Pragya S, Debebe M (2015). Dietary practices and associated factors among pregnant women in Wondo genet district, southern Ethiopia: a cross-section study. J Pharm Sci Innov.

[12] Zepro NB (2015). Food taboos and misconceptions among pregnant women of Shashemene district, Ethiopia, 2012. Sci J Public Health.

[13] Oluleke MO, Akintayo O, Ogunwale AO, Arulogun OS, Adelekan AL (2016). Dietary intake knowledge and reasons for food restriction during pregnancy among pregnant women attending primary health care centers in Ile-Ife, Nigeria. Int J Popul Stud.

[14] Getnet W, Aycheh W, Tessema T. Determinants of food taboos in the pregnant women of the Awabel District, East Gojjam Zone, Amhara Regional state in Ethiopia. Adv Public Health. 2018. 10.1155/2018/9198076.

[15] Meyer-Rochow VB (2009). Food taboos: their origins and purposes. J Ethnobiol Ethnomed.

[16] World Health Organization. Essential nutrition actions: Improving maternal, newborn, infant and young child health and nutrition. Geneva; 2013. https://app.who.int/iris/bitstream/handle/10665/84409/97892415055 (Accessed 21/07/2020) 25473713

[17] Kuzma J, Paofa D, Kaugla N, Catherina T, Samiak S, Kumei E (2013). Food taboos and traditional customs among pregnant women in Papua New Guinea: missed opportunity for education in antenatal clinics. Contemp PNG Stud.

[18] Tahir HMH, Ahmed AEEM, Mohammed NAA (2018). Food taboos among pregnant women in health centers, Khartoum state- Sudan, 2016. Int J Sci Healthc Res.

[19] Demissie K, Breckenridge MB, Rhoads GG (1998). Infant and maternal outcomes in the pregnancies of asthmatic women. Am J Respir Crit Care Med.

[20] Hartini TNS, Padmawati RS, Lindholm L, Surjono A, Winkvist A (2005). The importance of eating rice: changing food habits among pregnant Indonesian women during the economic crisis. Soc Sc Med.

[21] Arzoaquoi SK, Essuman EE, Gbagbo FY, Tenkorang EY, Soyiri I, Laar AK (2015). Motivations for food prohibitions during pregnancy and their enforcement mechanisms in a rural Ghanaian district. J Ethnobiol Ethnomed.

[22] Maimbolwa M, Ahmed Y, Diwan V, Arvidson AB (2003). Safe motherhood perspectives and social support for primigravidae women in Lusaka Zambia. Afr J Reprod Health.

[23] Puri S, Kapoor S (2006). Taboos and myths associated with women’s health among rural and urban adolescent girls in Punjab. Indian J Commun Med.

[24] M’soka NC, Mabuza LH, Pretorious D (2015). Cultural and health beliefs of pregnant women in Zambia regarding pregnancy and child birth. Curationis.

[25] Levay A, Mumtaz Z, Rashid SF, Willows N (2013). Influence of gender roles and rising food prices on poor, pregnant women’s eating and food provisioning in Dhaka, Bangladesh. Reprod Health.

[26] Mathews C, Benjamin V (1979). Health education evaluation of beliefs and practices in rural Tamil Nadu family planning and antenatal care. Soc Action.

[27] Rao M (1985). Food beliefs of rural women during the reproductive years in Dharwar, India. Ecol Food Nutr.

[28] Nag M (1994). Beliefs and practices about food during pregnancy. Econ Polit Wkly.

[29] Choudbry U (1997). Traditional practices of women from India: pregnancy, childbirth, and newborn care. Jognn.

[30] Manderson I (1987). Hot-cold and medical theories: overview and introduction. Soc Sci Med.

[31] Foster GM (1979). Humoral traces in United States folk medicine. Med Anthropol Newslett.

[32] Inam SNB, Punjani FZ, Omair A, Siddiqui S, Sultana S, Qureshi K. Beliefs of medical students in the hot and cold effects of food: impact of nutrition education. J Park Med Assoc. 2003;53(6):229–32.

[33] Foster GM (1987). The origin of humoral medicine in Latin America. Med Anthropol Quart.

[34] Lagay F. The legacy of humoral medicine. Virtual Mentor. 2002;4(7). 10.1001/virtualmentor.2002.4.7.mhst1-0207. 10.1001/virtualmentor.2002.4.7.mhst1-0207 23268770

[35] Rianga RM, Broerse JEW, Nangulu AK (2017). Food beliefs and practices among the Kalenjin pregnant women in rural Uasin Gish county, Kenya. J Ethnobiol Ethnomed.

[36] World Health Organization. Proper maternal nutrition during pregnancy planning and pregnancy: a healthy start in life. Geneva; 2017. http://www.euro.who.int/en/health.topics/diseaseprevention/nutrition/publications/2017/proper-maternal-nutrition-during-pregnancy-planning-and-pregnancy-a-healthy-start-in-life-2017 (Accessed 30/06/2020)

[37] World Health Organization. Nutrition and postpartum period. Geneva; 2006. edu/pwd/earn/firststeps/pdfs/mod5print.pdf (Accessed 30/06/2020)

[38] Cervera P, Ngo J (2001). Dietary guidelines for the breast-feeding woman. Public Health Nutr.

[39] Ihejiamaizu EC (2002). Issues in population policy and healthcare administration.

[40] Undelikwo VA, Enang EE (2018). Cultural practices and infant mortality in cross river state, Nigeria: A sociological perspective. Mediterr J Soc Sci.

[41] Kagiri LN, Mutuli LA, Bukhal P (2016). Socio-cultural practices and beliefs influencing infant and young child feeding in Lubao sub-location Kakamega country. J Nutr Health Food Eng.

[42] World Health Organization. Malnutrition. Geneva; 2020. Who.int/news-room/fact-sheets/detail/malnutrition (Accessed 01/07/2020)

[43] UNICEF. The state of the world’s children 2009: Maternal and newborn health. New York; 2008. Unicef.org/publications/files/SOWC_Spec.Ed_Main-Report-EN-090409.pdf (Accessed 25/07/2020)

[44] World Health Organization. Guiding principles for complementary feeding of the breastfed child. Geneva; 2001. https://whqlibdoc.who.int/paha/2003/a85622.pdf (Accessed 25/07/2020)

[45] Cleghorn G (2007). The role of red meat in meeting nutritional challenges during the life stages. Nutr Diet.

[46] Emily M. Relationship between breastfeeding practices and nutritional status of children aged 0-24 months in Nairobi, Kenya. Afr J Food Agric Nutr Dev. 2010;10(4):2358–78.

[47] Davison AN, Bobbing J (1966). Myelination as a vulnerable period in brain development. Brit Med Bull.

[48] Faber M, Benade AJS (2007). Breastfeeding, complementary feeding and nutritional status of 6-12-month-old infants in rural KwaZulu-Natal. South Afr J Clin Nutr.

[49] Du Plessis LM, Sweet L (2013). Complementary feeding: a critical window of opportunity from six months onwards. South Afr J Clin Nutr.

[50] Bishnoi I, Gupta S, Gupta JNP (1994). Traditional beliefs and practices regarding nutrition during pregnancy. Indian J Nutr Diet.

[51] Ali NS, Azam SI, Noor R (2004). Women’s belief and practices regarding food restrictions during pregnancy and lactation: a hospital based study. J Ayub Med Coll Abbottabad.

[52] Shahid A, Khan MW, Ahmed M, Rehman M, Rashid F (2011). Women beliefs and practices regarding food during pregnancy. A hospital based study. Prof Med J.

[53] Hossain B, Sarwar T, Reja S, Akter MN. Nutritional status of pregnant women in selected rural and urban area of Bangladesh. J Nutr Food Sci. 2013;3(4). 10.4172/2155-9600.1000219.

[54] Oluwafolahan OS, Catherine AB, Olubukunola AJ (2014). Dietary habits of pregnant women in Ogun-East senatorial zonem Ogun State, Nigeria: a comparative study. Int J Nutri Metab.

[55] Kuche D, Singh P, Moges D (2015). Dietary practices and associated factors among pregnant women in Wond genet district, southern Ethiopia: a cross-section study. J Pharm Sci Innov.

[56] Guindeuli T (2014). What do Christians (not) eat: food and Ethiopian Christian communities (12th – 18th C). Annales d’Ethiopie.

[57] Tela FG, Gebremariam LW, Beyene SA (2020). Food taboos and related misperceptions during pregnancy in Mekelle city, Tigray, northern Ethiopia. Plos one.

[58] Zinyemba L (2020). Understanding maternal health care through the role played by dietary food taboos in Binga. Afr J Soc Work.

[59] Umhlabuyalingana municipality integrated development plant 2011-2016, 2020. Umhlabuyalingana.gov.za/wp-content/uploads/2020/07/Reviewed-Final-IDP-2020-Repaired.pdf (Accessed 13/11/2020).

[60] Liamputtong P, Yimyam S, Parisunyakul S, Baosoung C (2005). Sanisiriphun. Traditional beliefs about pregnancy and child birth among women from Chiang Mai, northern Thailand. Midwifery.

[61] Liu N, Mao L, Sun X, Liu L, Chen B, Ding Q (2006). Postpartum practices of puerperal women and their influencing factors in three regions of Hubei, China. BMC Public Health.

[62] Turner C, Pol S, Suon K, Neou L, Day NPJ, Parjer M, Kingori P (2017). Beliefs and practices during pregnancy, post-partum and in the first days of an infant’s life in rural Cambodia. BMC Pregnancy Childbirth.

[63] McNamara K, Wood E (2019). Food taboos, health belief, and gender: Understanding household food choice and nutrition in rural Tajikistan. J Health Popul Nutr.

[64] Ngunyulu RN, Mulaudzi FM (2009). Indigenous practices regarding postnatal care at Sikhunyani village in Limpopo province of South Africa. South Afr J Nurs Midwifery.

[65] Chakona G, Shackleton C (2019). Food taboos and cultural beliefs influence food choice and dietary preferences among pregnant women in the Eastern Cape, South Africa. Nutrients.

[66] Ngomane S, Mulaudzi FM. Indigenous beliefs and practices that influence the delayed attendance of antenatal clinics by women in the bohlabelo district in Limpopo, South Africa. Midwifery. 2012. 10.1016/j.midw.2010.11.002. 10.1016/j.midw.2010.11.002 21146264

[67] Bopape MM, Mbhenyane XG, Alberts M (2008). The prevalence of anemia and selected micronutrients status in pregnant teenagers of Polokwane municipality in Limpopo province. South Afr J Clin Nutr.

[68] Mmusi-Phetoe RMM (2016). Social factors determining maternal and neonatal mortality in South Africa: A qualitative study. Curationis.

[69] Symington EA, Baumgarter J, Malan L, Zandberg L, Ricci C, Smuts CM (2018). Nutrition during pregnancy and early development (NuPED) in urban South Africa: a study protocol for a prospective cohort. BMC Pregnancy Childbirth.

[70] Napier C, Warriner K, Sibiya MN, Reddy P (2019). Nutritional status and dietary diversity of pregnant women in rural KwaZulu-Natal, South Africa. Health SA.

[71] Chakona G (2020). Social circumstances and cultural beliefs influence maternal nutrition, breastfeeding and child feeding practices in South Africa. Nutr J.

[72] Downie D, Antipatis C, Delday MI, Maitin CA, Sneddo AA (2005). Moderate maternal vitamin A deficiency alters myogenic regulatory protein expression and perinatal organ growth in the rat. Am J Physiol Regul Integr Comp Physiol.

[73] World Health Organization. Vitamin A supplementation in pregnant women. Geneva; 2011. https://apps.who.int/irs/bitstream/handle/10665/4462/9789241501781_eng.pdf?ua=1 (Accessed, 10/08/2020)

[74] Casanueva E, Ripol C, Tolentino M, Morales RM, Pfeffer F, Vilchis P, Vadillo-Ortego F (2005). Vitamin C supplementation to prevent premature rupture of the choriamniotic membranes: a randomized trial (1-3). Am J Clin Nutr.

[75] Ningtyias FW, Kurrohman T. Food taboos and recommended foods for pregnant women: the study of phenology in Pendhalungan society. Earth Environ Sci. 2020;485. 10.1088/1755-1315/485/1/012149.

[76] Lannotti LL, Lutter CK, Bunn DA, Stewart CP (2014). Eggs: The untracked potential for improving maternal and young child nutrition among the world’s poor. Nutr Rev.

[77] Zeisel HH, Niculescu M (2006). Perinatal choline influences brain structure and function. Nutr Rev.

[78] Trumpff C, Vandevijvere S, Moreno-Reyer R, Vanderpas J, Tafforeau J, Van Oyen H, De Schepper J (2015). Neonatal thyroid-stimulating hormone level is influenced by neonatal, maternal and pregnancy factors. Nutr Res.

[79] Moffatt CRM, Musto J. S*almonella* and egg-related outbreaks. Microbiol Aust. 2013. 10.1071/MA13033.

[80] Duguid JP, North RAE (1991). Eggs and *Salmonella* food poisoning: an evaluation. J Med Microbiol.

[81] Whiley H, Ross K (2015). *Salmonella a*nd eggs: from production to plate. Int J Environ Public Health.

[82] Chakrabarty S, Mominul AKM, Aminul Isla AKM (2017). Nutritional benefits and pharmaceutical potentialities of chili: A review. Fundam Appl Agric.

[83] Payan DG, Levin JD, Goetzl EJ (1984). Modulation of immunity and hypersensitivity by sensory neuropeptide. J Immunol.

[84] Kiran DKA, Roberson J, Geraghty DP, Ball MJ (2006). Effects of chill consumption of postprandial glucose, insulin and energy metabolism. Am J Clin Nutr.

[85] Wead WB, Cassidy SS, Coast JR, Hagler HK, Reynold RC (1987). Reflex cardiorespiratory responses to pulmonary vascular congestion. J Appl Physiol.

[86] Sontag C, Ketaneh A, Fain O, Eclache V, Thomas M (2001). Rapid regression of prolonged pagophagia after treatment of iron deficiency. Presse Med.

[87] Holmes W, Hoy D, Lockley A, Thammavonyxay K, Bounnaphol S, Xeuatvongsa A, Toole M (2007). Influences on maternal and child nutrition in the highlands of the northern Lao PDR. Asia Pac J Clin Nutr.

[88] Goran MI, Plows JF, Ventura EE (2019). Effects of consuming sugars and alternative sweeteners during pregnancy on maternal and child health: evidence for a secondhand sugar effect. Proc Nutr Soc.

[89] Bedard A, Northstone K, Shaheen SO (2017). Maternal intake of sugar during pregnancy and childhood respiratory and atopic outcomes. Eur Respir J.

[90] Shin D, Lee KW, Song WO (2015). Dietary patterns during pregnancy are associated with the risk of gestational diabetes mellitus. Nutrients.

[91] Popova S, Rehm J (2017). Prevalence of alcohol consumption during pregnancy and fetal alcohol spectrum disorders among the general and aboriginal populations in Canada and United States. Eur J Med Genet.

[92] FASD (2012). The hidden harm-inquiry into the prevention, diagnosis and management of fetal alcohol spectrum disorders.

[93] Bhuvaneswar CJ, Chang G, Stern TA (2007). Alcohol use during pregnancy: prevalence and impact. Prim Care Companion J Clin Psychiatry.

[94] Alwan NA, Cade JE, McArdle HJ, Greenwood DC, Hayes HE, Simpson NA (2015). Maternal iron status in early pregnancy and birth outcomes: insights from the baby’s vascular health and iron in pregnancy study. Br J Nutr.

[95] Hoey L, McNulty H, Duffy ME, Hunges CF, Strain JJ (2013). EURRECA-Estimating folate requirements for deriving dietary reference values. Crit Rev Food Sci Nutr.

[96] Maia SB, Souza ASR, Caminha MFC, Da Silva SL, Cruz RRC, Dos Santos CC, Filho MB (2019). Vitamin A and pregnancy: a narrative review. Nutrients.

[97] Institute of Obstetricians and Gynecologists Royal College of Physicians of Ireland and Directorate of Clinical Strategy and Programmes, Health Service Executive. Nutrition for pregnancy. Clin Pract Guideline. 2016; hse.ie/eng/services/publications/clinical-strategy-and-programmes/imews-guidelines-pdf.

[98] Taylor CM, Emmett PM, Emond AM, Golding J (2018). A review of guidance on fish consumption in pregnancy: Is it fit for purpose?. Public Health Nutr.

[99] Zimmermann MB (2011). The role of iodine in human growth and development. Semin Cell Dev Biol.

[100] Chen Z, Myers R, Wei T, Bind E, Kassim P, Wang G, Ji Y, Hong X, Caruso D, Bartell T, Gong Y, Strickland P, Navas-Acien A, Guallar E, Wang X (2014). Placenta transfer and concentration of cadium, mercury, lead and selenium in mothers, newborns and young children. J Expo Sci Environ Epidemiol.

[101] Starling P, Charlton K, McMahon AT, Vucas C (2015). Fish intake during pregnancy and foetal neurodevelopment—a systematic review of the evidence. Nutrients.

[102] Moreno MA, Furtner F, Rivara FP (2012). Exposure to mercury and consumption of fish during pregnancy: a confusing picture. Arch Pediatr Adolesc Med.

[103] Faber M (2005). Complementary food consumed by a 6-12-month-old rural infants in South Africa as inadequate in micronutrients. Public Health Nutr.

[104] Stefanello J, Nakano AM, Gomes FA (2008). Beliefs and taboos related to the care after delivery: their meaning for a women group. Acta Paul Enferm.

[105] Nolte AGW (2011). A textbook for midwives.

[106] Rauha JP, Remes S, Heinone M, Hopia A, Kahkonen M, Kujala T, Vuorela H, Vuorela P (2005). Antimicrobial effects of Finnish plant extracts containing flavonoids and other phenolic compounds. Int J Food Microbiol.

[107] Al-Aboud NM (2008). Effects of red beetroot (*Beta vulgaris* L.) intake on the level of some hematological tests in a group of female volunteers. ISABB J Food Agric Sci.

[108] Ogbeide O (1974). Nutritional hazards of food taboos and preferences in Mid-West Nigeria. Am J Clin Nutr.

[109] Allen LH, Gillespie SR (2001). What works? A review of the efficacy and effectiveness of nutrition interventions.

[110] Dewey KG, Brown KH (2003). Update on technical issues concerning complementary feeding of young children in developing countries and implications for intervention programs. Food Nutr Bull.

[111] Algarin C, Peirano P, Garrido M, Pizarro F, Lozoff B (2003). Iron deficiency anemia in infancy: Long lasting effects on auditory and visual system functioning. Pediatr Res.

[112] Natasa F, Braegger RC, Bronsky J, Campoy C, Domellof M, Embleton ND, Hojisak I, Hulst J, Indrio F, Lapillonne A, Mihatsch W, Molgaard C, Vora R, Fewtrell M (2017). Sugar in infants, children and adolescents: a position paper of the European Society for Paediatric Gastroenterology, Hepatology and Nutrition. JPGN.

[113] Johnson RK, Apper LJ, Brands M, Howard BV, Lefevre M, Lustig RH, Sacks F, Steffen LM, Wylie-Rosett J (2009). Dietary sugar intake and cardiovascular health: a scientific statement from American heart association. Circulation.

[114] Mounder EMW, Nel JH, Steyn NP, Kruger HS, Labadarious D (2015). Added sugar, macro- and micronutrient intake and anthopometry of children in a developing world context. PLOS One.

[115] Marais NC, Christofides NJ, Erza A, Hofman KJ (2019). Evidence for high sugar content of baby foods in South Africa. South Afr Med J.

